# Determinants of host feeding success by *Anopheles farauti*

**DOI:** 10.1186/s12936-016-1168-y

**Published:** 2016-03-10

**Authors:** Tanya L. Russell, Nigel W. Beebe, Hugo Bugoro, Allan Apairamo, Robert D. Cooper, Frank H. Collins, Neil F. Lobo, Thomas R. Burkot

**Affiliations:** Australian Institute of Tropical Health and Medicine, James Cook University, Cairns, QLD 4870 Australia; School of Biological Sciences, University of Queensland, St. Lucia, QLD 4068 Australia; CSIRO, Dutton Park, Brisbane, QLD 4102 Australia; National Vector Borne Disease Control Programme, Ministry of Health, Honiara, Solomon Islands; Australian Army Malaria Institute, Gallipoli Barracks, Enoggera, 4052 Australia; Eck Institute for Global Health, Department of Biological Sciences, University of Notre Dame, Notre Dame, IN 46556 USA

**Keywords:** Host fidelity, Mark-release-recapture, Human blood index (HBI), Barrier screens, Outdoor resting, Fecundity, *An. farauti*, Solomon Islands

## Abstract

**Background:**

The proportion of blood meals that mosquitoes take from a host species is a function of the interplay of extrinsic (abundance and location of potential hosts) and intrinsic (innate preference) factors. A mark-release-recapture experiment addressed whether host preference in a population of *Anopheles farauti* was uniform or if there were anthropophilic and zoophilic subpopulations. The corresponding fitness associated with selecting different hosts for blood meals was compared by measuring fecundity.

**Methods:**

The attractiveness of humans for blood meals by *An. farauti* in the Solomon Islands was compared to pigs using tent traps. Host fidelity was assessed by mark-release-recapture experiments in which different colour dusts were linked to the host to which the mosquito was first attracted. Outdoor resting *An. farauti* were captured on barrier screens and the human blood index (HBI) as well as the feeding index were calculated. The fecundity of individual *An. farauti* after feeding on either humans or pigs was assessed from blood-fed mosquitoes held in individual oviposition chambers.

**Results:**

*Anopheles farauti* were more attracted to humans than pigs at a ratio of 1.31:1.00. The mark-release-recapture experiment found evidence for *An. farauti* being a single population regarding host preference. The HBI of outdoor resting *An. farauti* was 0.93 and the feeding index was 1.29. *Anopheles farauti* that fed on a human host laid more eggs but had a longer oviposition time compared to *An. farauti* that had blood fed on a pig.

**Conclusions:**

One of the strongest drivers for host species preference was the relative abundance of the different host species. Here, *An. farauti* have a slight preference for humans over pigs as blood meal sources. However, the limited availability of alternative hosts relative to humans in the Solomon Islands ensures a very high proportion of blood meals are obtained from humans, and thus, the transmission potential of malaria by *An. farauti* is high.

**Electronic supplementary material:**

The online version of this article (doi:10.1186/s12936-016-1168-y) contains supplementary material, which is available to authorized users.

## Background

Malaria transmission is governed by the interactions between humans, *Plasmodium* parasites and the anopheline mosquito vectors. This is mathematically expressed by the Ross-MacDonald model [1]. The parameter representing a mosquito’s propensity to feed on humans is exponentially related to malaria transmission so small changes in human feeding rates will have substantial impacts on transmission potential. Crucially, mosquitoes differ in their tendency to feed on human or animal blood by species and also within species across different geographic areas or villages [2–5]. Despite this variation, the preference to feed on a particular host species cannot be attributed simply to random foraging but is influenced by the interplay of both extrinsic and intrinsic factors [4, 6]. These factors include the nutritional value of the host blood, the relative abundance of potential host species (a function of absolute numbers, locations [including house construction], defensive behaviours), and innate/genetic factors that may regulate plasticity (e.g., the ability of individual mosquitoes to switch or maintain host choice).

The properties of hosts that influence their attractiveness to mosquitoes include odorants, body heat and body mass [4, 6]. Host blood has a direct selection feedback in terms of the potential survival of individual mosquitoes and reproductive fitness as expressed by fecundity [6–8]. The relative abundance and accessibility of different hosts (i.e. nature of housing, access to bed nets or other factors that make a host more or less available) also influences the feeding rate on hosts. For example, where cattle are kept close to humans, the human blood index (HBI) of the zoophilic *Anopheles arabiensis* is reduced [9, 10]. In Papua New Guinea, modifications in house construction and animal husbandry practices resulted in changes in the proportion of blood meals by *An. farauti* on humans, pigs and dogs [11]. Mosquitoes sampled resting inside houses tend to have higher HBIs relative to outdoor collected samples [4, 5, 12]. As such, when monitoring the host preferences of a mosquito population it is important to obtain an unbiased sample of blood fed resting mosquitoes [13, 14] and also to standardize the observed HBI against the relative proportions of the available hosts in the environment [15, 16].

Several studies observed that mosquitoes return to feed on their original host species at higher than expected rates [17–22], however the basis for observed host fidelity by mosquitoes remains unclear. Evidence for learning by mosquitoes has been questioned due to the absence of appropriate controls or replication [23, 24]. Genetic polymorphism in host preference was demonstrated for *An. gambiae* in the 1960s, with preferences for humans or cows selected for within 5–6 generations in Tanzania [22]. During the 1970s, genetic control of host preference for *Aedes simpsoni* and *Aedes aegypti* was demonstrated by backcrossing strains of these species resulting in hybrids with a host preference intermediate to the parental strains [25]. Host preference was also shown to be associated with chromosomal inversion polymorphisms in *An. arabiensis* from Ethiopia [26] and Kenya [27].

The current study examined the influence of these factors on the host preferences of the primary malaria vector, *An. farauti,* in Melanesia which varies in its degree of anthropophagy across the region [5]. Specifically, experiments elucidated the relative attractiveness of *An. farauti* to different host species as well as feeding success. The fidelity of individual mosquitoes to be repeatedly attracted to the same host species (humans or pigs) was examined by mark-release-recapture experiments to determine if subpopulations of anthropophilic or zoophilic mosquitoes exist. This information was linked to data on the relative rates of blood feeding on the different available host species in a village by measuring the human blood index and zoophilic blood indices. Mosquito fitness as defined by fecundity per feeding cycle was ascertained after *An. farauti* had obtained blood meals on either humans or pigs.

## Methods

### Study site

The study was conducted in Haleta village on Ngella Sule Island in Central Province, Solomon Islands (–9°5′56″S, 160°6′56″E) [28], a coastal village with 470 residents (Bed net census, 2010, Ministry of Health, Unpublished data). In August 2012 there were 40 pigs (21 adults and 19 piglets) in the village. The climate is hot and wet with an annual rainfall of 2837 mm (based on 43 years of data collected at the provincial capital Tulagi approximately 10 km from Haleta village) [29]. The mean annual temperature is 26 °C. *Anopheles farauti* sensu stricto is the dominant malaria vector in the Solomon Islands and the only known vector in Haleta village [28].

### Relative attractiveness of hosts

**Fig. 1:**
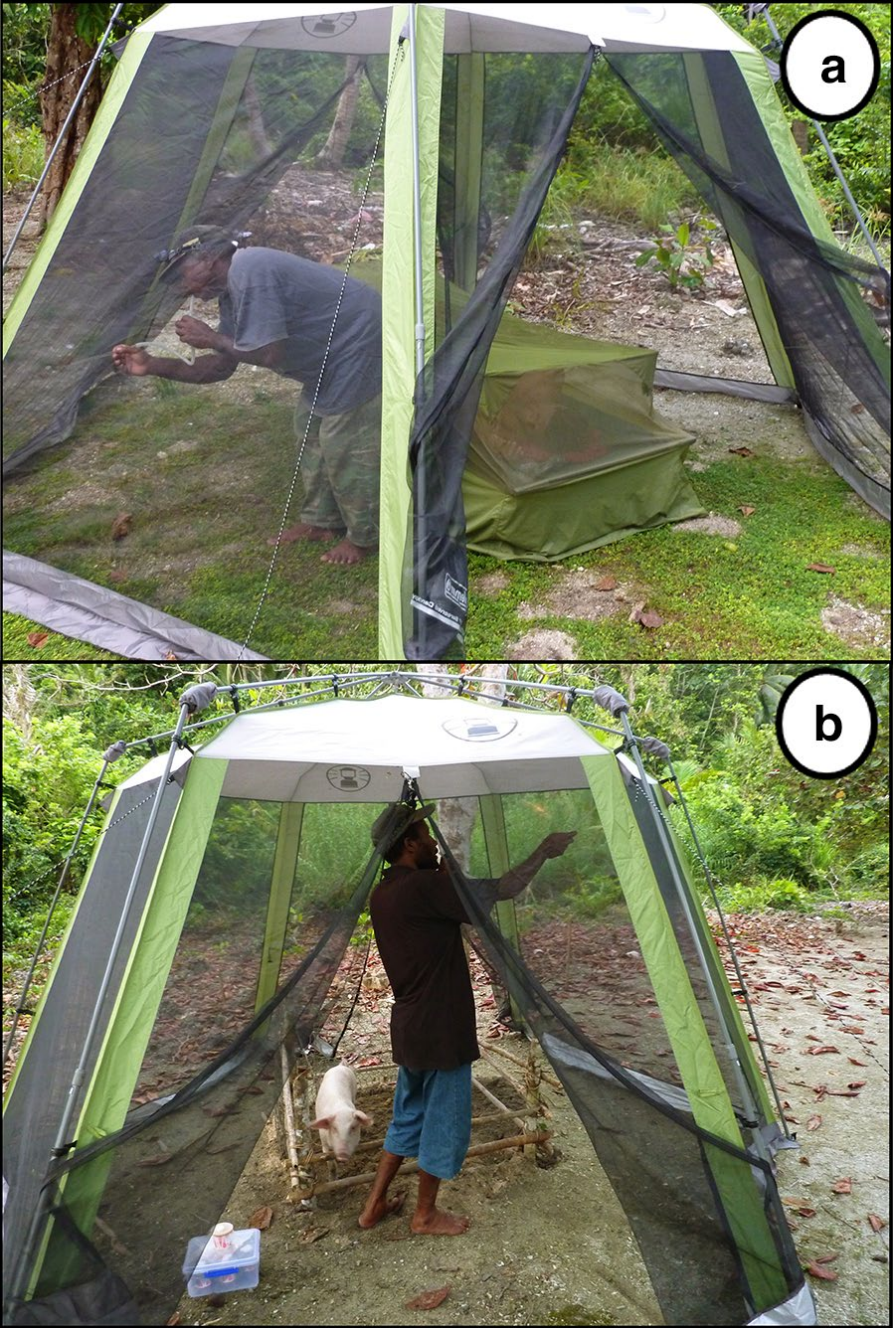
Animal baited tents with (**a**) human and (**b**) pig hosts

The relative attractiveness of *An. farauti* to humans and the dominant domestic animal (the pig) in Haleta village was determined over 14 consecutive nights from 31st July–13th August 2012 using host baited traps to attract and capture anophelines. The traps consisted of tents (Coleman Hexagonal Screened Canopy, Model No. 2000004414) erected over either a human resting on a cot under a bed net or a pig confined within a pen. Both doors of the tents were pinned open to facilitate mosquito entry (Fig. 1). Five replicate pairs of traps were constructed: five tents housed humans resting on cots under bed nets and five tents housed pigs in their pens. The traps were constructed along the length of the village [28], with hosts alternating between pigs and humans. Adjacent traps were a minimum of 20 m apart. Pigs were cared for by their owners as per their usual routine to prevent unnecessary stress.

Village mosquito collectors entered the tents hourly between 18.00–00.00 h to capture mosquitoes resting inside tent walls with a mouth aspirator. Collectors wore mosquito repellent and stayed >10 m from the traps when not collecting resting mosquitoes to minimize luring mosquitoes into the traps by the presence of the collectors. The people acting as baits and the collectors were rotated among each of the stations nightly to minimize biases due to individual host attractiveness. The efficacy of the animal baited tents for attracting and capturing mosquitoes was compared directly with human landing catches (HLC) conducted simultaneously. The HLC was conducted by four collectors stationed outdoors and positioned between the tents from 1800–00.00 h.

### Host fidelity

A mark-release-recapture experiment examined if individual *An. farauti* were repeatedly attracted to the same host species (e.g., if a mosquito seeks a blood meal on a human, will it return to feed on a human in the next feeding cycle or is host selection flexible). *Anopheles farauti* captured in the tent trap experiment, detailed above were the source of mosquitoes used in this experiment. During the first 10 nights of the experiment, at hourly intervals all *Anopheles* were morphologically identified, offered a blood meal on the host species to which they were originally attracted (according to the approved Institutional Review Board protocols, see "Ethics" section), and counted. At 00.00 h, all blood fed mosquitoes were marked with fluorescent dusts and released between midnight and 01.00 h. Mosquitoes caught resting in the human-baited tent were marked with one colour of fluorescent dust and mosquitoes captured resting in pig-baited tents were marked with a different colour dust before release. On nights 11–14 of the experiment, resting mosquitoes were collected in the tents, as described above, but not released. All *Anopheles* captured on nights two through 14 were visually checked for fluorescent dust using a UV torch. Recaptured mosquitoes (e.g., marked with fluorescent dust) were not released again.

For marking, a maximum of 100 blood-fed mosquitoes were placed into plastic 250 ml cups covered with netting. A small amount of fluorescent powder (BioQuip Products, Inc. California, USA and Glow Paint Industries, Queensland, Australia) was sifted through the netting into the cup; a fine tipped transfer pipette was used to aerosolize the powder which adhered to the mosquitoes. The effectiveness of this marking procedure was checked by examining the mosquitoes in each cup with a LED UV torch (400 nm wavelength) to ensure adequately marking. The mosquitoes were released on the night of collection from a single outdoor location. The distance from the release site to the furthest collection station was 190 m.

### Human blood index (HBI) of outdoor resting mosquitoes

**Fig. 2:**
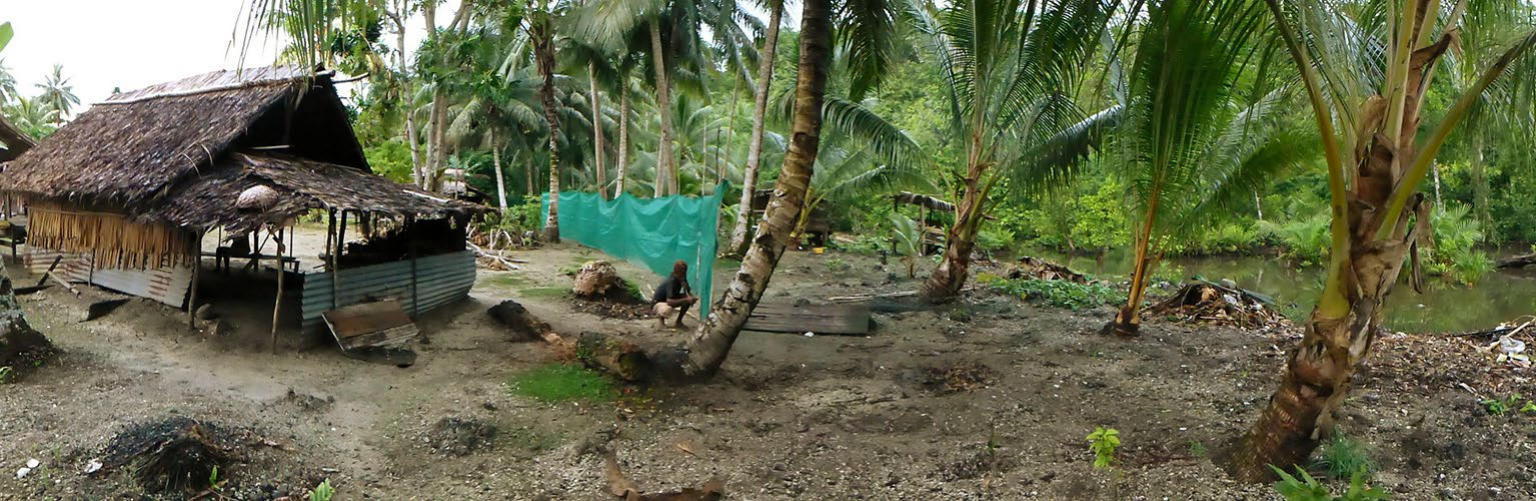
Outdoor resting mosquitoes were sampled using a barrier screen which was constructed between village houses (*left*) and potential resting and/or oviposition sites (*right*)

Outdoor resting mosquitoes were collected using barrier screen traps [14] over 23 months from August 2011–February 2014. During this time, simultaneous collections of host seeking mosquitoes were made using HLC (see [28]). Four 20 m long barrier screens were constructed from 2 m high polyethylene shade cloth (70 % shading) secured to wooden poles at 2 m intervals with polyester cord. The barrier screens were constructed to intercept mosquitoes flying between the village (where blood meals on humans and domestic animals could be obtained) and resting sites (e.g., bush areas close to a permanent swamp, the dominant oviposition site; Fig. 2). Mosquitoes resting on the barrier screens were captured with a mouth aspirator. Each side of the barrier screen was searched for approximately 20 min per hour. Mosquito collections were conducted between 18.00–00.00 h, except during 23rd Nov–6th Dec 2011 when collections were made between 18.00–06.00 h. Mosquitoes captured on each side of the barrier screen were stored separately. All mosquitoes were morphologically identified to sex and species [30]. Resting mosquitoes on the barrier screens were visually classified as being unfed, partially fed, fully fed, gravid or sugar fed.

### Fecundity associated with blood meals on different host species

The fecundity of *An. farauti* was determined directly from blood-fed mosquitoes which had fed on different host species. Pig-fed mosquitoes were captured inside tents (described above) in which the only blood source was a pig or unfed mosquitoes were released into a sealed tent containing a pig at midnight and engorged mosquitoes retrieved at 06.00 h. Human fed *An. farauti* were obtained from HLC collections.

Individual blood-fed *An. farauti* were held in oviposition chambers constructed from 70 ml plastic specimen jars holding a piece of damp cotton-wool covered with filter paper on the bottom as an oviposition substrate. The top of each oviposition chamber was covered with mosquito netting overlaid with damp cotton-wool to ensure high humidity. Each oviposition container was examined daily and the number of eggs laid was recorded.

### Laboratory analysis

Specimens were stored in 100 % ethanol in micro-centrifuge tubes. A sample of the captured *An. farauti* was identified by molecular analysis of the internal transcribed spacer region II of the ribosomal DNA [31]. Host blood meal sources (human, pig and dog) for *An. farauti* were identified by PCR using slight modifications of the Kent and Norris method [32]. The PCR reactions consisted of 0.4 μM of each primer, 3.0 mM MgCl_2_, 1.0 mM dNTPs and 0.5 units of Taq polymerase, with a final volume of 25 μl for each reaction. The gut content of the females captured resting outdoors was analysed disregarding visual classification of abdominal status, so as to include specimens which contained trace amounts of blood that weren’t visually evident [33].

### Statistical analysis

The data was compiled in a series of tables which detailed the results of: 1. mosquito collections, 2. molecular analyses, 3. mark-release-recapture releases, and 4. oviposition [34]. Generalized linear models (GLMs) with a negative binomial distribution and a categorical explanatory variable for trap type compared: (1) the efficacy of the human baited tent traps for catching mosquitoes compared with HLC; and (2) the relative attractiveness of humans and pigs to *An. farauti*. The results of the mark-release-recapture experiment were analysed using a GLM with a binomial distribution, a categorical explanatory variable for mosquito label (i.e. unmarked or dusted) and a dependent binary variable containing the number of mosquitoes caught in either human or pig baited tents. Lastly, the fecundity of *An. farauti* was investigated with a Poisson GLM with explanatory variables for host species and the number of nights taken to oviposit with an interaction term. All analyses were conducted using the *R* package V3.1.2 [35].

The feeding index (FI), the proportion of feeds on one host with respect to another divided by the expected comparative proportion of feeds on those two hosts, was calculated [16] as (Ne/Ne′)/(Ef/Ef′); where Ne and Ne′ = the number of feeds on host I (humans) and host II (pigs), respectively; and Ef and Ef’ = the density of host I and host II, respectively.

### Ethics

Ethical approval for the study was obtained from the National Health Research and Ethics Committee, Solomon Islands (02-05-2011), the James Cook University Human Research Ethics Committee, Australia (H4122) and Animal Ethics Committee (A1616), the University Hospitals Case Medical Centre Institutional Review Board for Human Investigation, USA (05-11-11). Mosquito collectors were recruited from the village residents after the risks were explained and they signed an informed consent agreement. Only village adults who likely have some immunity to malaria were asked to participate in the landing catches and were instructed to capture the mosquitoes before they bite and all took malaria prophylaxis. For the host fidelity mark-release-recapture experiment, mosquitoes were offered a blood meal on the host species to which they were originally attracted; for the human-attracted mosquitoes blood meals were obtained from one of the listed authors who was taking malaria prophylaxis prior to release.

## Results

### Relative attractiveness of hosts

**Fig. 3:**
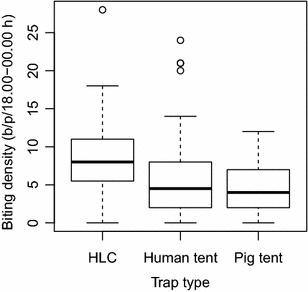
Comparison of the densities of *Anopheles farauti* caught with human landing catch (HLC) and in human-baited tents in Haleta village, Central Province, Solomon Islands. Note b/p/18.00–00.00 h = bites/person/18.00–00.00 h

**Fig. 4:**
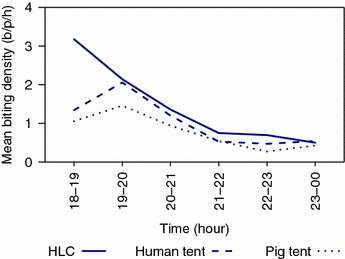
The hourly profile of *Anopheles farauti* biting humans and caught in animal-baited tents in Haleta village, Central Province, Solomon Islands. Note b/p/h = bites/person/hour

During the 14 night experiment, the number of *An. farauti* caught by HLC was 483 (median = 8.0, first quartile [Q1] = 5.5, third quartile [Q3] = 11.0), with 430 captured in human-baited tents (median = 4.5, Q1 = 2.0, Q3 = 8.0) and 329 in pig-baited tents (median = 4.0, Q1 = 2.0, Q3 = 7.0). All specimens (n = 87) were confirmed as *An. farauti s.s.* by molecular analysis. The density of mosquitoes caught per human tent was slightly lower than that caught with outdoor HLC (Fig. 3; Relative ratio = 0.712 ± 0.129, *p* = 0.009). Fewer *An. farauti* were captured in tents during the first hour of the night compared to HLCs (Fig. 4). The ratio of mosquitoes caught in the human-baited tents compared to pig-baited tents was 1.31:1.00, indicating that *An. farauti* are slightly but statistically significantly more attracted to humans than pigs (Relative ratio = 0.765 ± 0.131; *p* = 0.041).

### Host fidelity

**Fig. 5:**
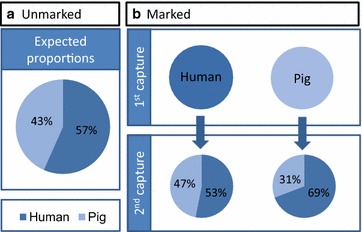
The relative attractiveness (**a**) and fidelity (**b**) of attraction to different animal species by *Anopheles farauti* as determined from a mark-release-recapture experiment

To assess host fidelity with mark-release-recapture a total of 457 marked *An. farauti* were released (202 initially caught in pig-baited tents and 255 initially caught in human-baited tents), and 9.8 % (n = 45) were recaptured in the tent traps. Of the marked *An. farauti* released after initial capture in human-baited tents, 53 % (n = 17) were recaptured in human-baited tents, with 47 % (n = 15) being recaptured from pig-baited tents. Of the mosquitoes that were originally captured in pig-baited tents, 69 % (n = 9) were recaptured from human-baited tents and 31 % (n = 4) were recaptured in pig-baited tents (Fig. 5). The ratio of *An. farauti* recaptured in human and pig-baited tents was not significantly different from the proportion of unmarked *An. farauti* that were originally attracted to either humans or pigs (Table 1), indicating that *An. farauti* is a single population with regards to host fidelity.

**Table 1:** Analyses by binomial generalized linear model (GLM) of the proportion of *Anopheles farauti* caught inside human-baited or pig-baited tents in comparison to the relative attractiveness of each host as determined from the numbers captured in each host-baited tent trap

Label of captured mosquitoes	Proportion caught in human-baited tent (n/total)	Odds ratio (se)	*p* value
Unmarked	0.57 (404/714)		
Released from human-baited tent	0.53 (17/32)	0.870 (0.362)	0.700
Released from pig-baited tent	0.69 (9/13)	1.726 (0.606)	0.367

### Human blood index (HBI) of outdoor resting mosquitoes

**Fig. 6:**
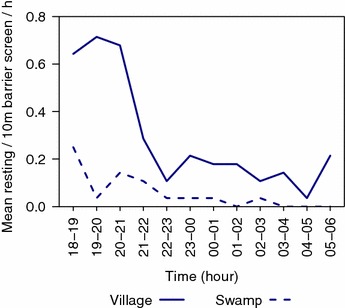
The hourly profile of *Anopheles farauti* resting on barrier screens in Haleta village, Central Province, Solomon Islands, compared for the two sides of the barrier screen which faced either the village houses (labelled village) and potential resting and/or oviposition sites (labelled swamp)

A total of 1882 female and 178 male *An. farauti* were collected resting on the barrier screens. All female specimens examined (n = 1059) were confirmed as *An. farauti s.s.* by molecular analysis. Seasonal fluctuations in the density of resting mosquitoes were observed, and the temporal fluctuations mirrored the biting densities recorded using HLC (Additional file 1). The majority of female *An. farauti* (78 %; n = 91/117) were caught resting on the barrier screen before midnight (Fig. 6) and were mostly (69 %; n = 1298) on the village side of the fence where blood meals would be more readily available compared to the opposite side where oviposition and resting sites predominate (31 %; n = 584). The abdominal status of 1498 females captured on the barrier screens were 56.3 % (n = 844) unfed, 42.2 % (n = 632) blood fed, 0.4 % (n = 6) gravid and 1.1 % (n = 16) sugar fed. Host blood meal source analysis of 1312 specimens was conducted and 774 were confirmed to be unfed. Of the 538 blood fed *An. farauti*, 91 % (n = 492) contained only human blood, 4 % (n = 24) contained pig blood, 1 % (n = 6) contained dog blood, 2 % (n = 9) contained mixed human and pig blood meals and 1 % (n = 7) contained unidentified blood, giving an HBI for *An. farauti* of 0.93.

### Feeding index

The feeding index is a calculation of the relative feeding preference on two host species based on the feeding success on each of two hosts compared to the abundance of the same two host species. The ratio of the number of feeds by *An. farauti* on humans (Ne) and pigs (Ne′) was 15.18 (501/33) as obtained from the blood meal analyses of resting *An. farauti*. The ratio of the number of humans (Ef) and pigs (Ef′) as determined by a census survey was 11.75 (470/40). The feeding index was 1.29 (15.18/11.75) indicating a slight preference of *An. farauti* to feed on humans rather than pigs.

### Fecundity after feeding on different hosts

The mean number of eggs laid after feeding on a human was 117.3 ± 3.5 (n = 154) and after feeding on a pig was 104.6 ± 7.8 (n = 42) (Table 2). When *An. farauti* fed on a human host, they laid significantly more eggs but took longer to oviposit, with the majority of oviposition events occurring on the second and third nights after feeding (Table 2). When *An. farauti* fed on a pig, fewer eggs were laid but oviposition occurred sooner (e.g., on the second night after blood feeding). The interaction term between host and time to oviposition in the GLM was significant (Table 3), as there was an opposing relationship between fecundity and time to oviposit for each host. For human-fed *An. farauti*, the longer time for egg production was associated with higher egg production whereas, pig-fed *An. farauti*, produced fewer eggs albeit with a shorter oviposition interval.

**Table 2:** Oviposition length and fecundity of *Anopheles farauti* after feeding on different host species

No of nights since feeding	Proportion ovipositing (n) by host		Mean number of eggs	
	Human host	Pig host	Human host	Pig host
1	0.00 (0)	0.03 (1)	NA	105.0
2	0.44 (64)	0.78 (28)	103.5	105.0
3	0.46 (68)	0.17 (6)	118.0	71.7
4	0.10 (14)	0.03 (1)	127.6	23.0
Overall	1.00 (164)	1.00 (36)	114.8	97.1

**Table 3:** The effect of host blood meal source and length of egg development on fecundity of *Anopheles farauti* analysed with a generalized linear model (GLM)

Experimental factor	*β* (se)	*p* value
Host	0.948 (0.091)	<0.0001
Nights taken to oviposit	0.108 (0.015)	<0.0001
Interaction term	−0.483 (0.040)	<0.0001

## Discussion

In this study, four major aspects of blood feeding by *An. farauti* were examined: reproductive fitness associated with host feeding (characterized by fecundity), host attractiveness (defined by host choice experiments and calculations of feeding indices), human host-feeding success (measured by the HBI) and fidelity of host selection (characterized by mark-release-recapture experiments). A potential factor driving host blood meal choice in mosquitoes is reproductive fitness as expressed by fecundity [6]. In this study in the Solomon Islands, *An. farauti* was slightly more attracted to humans than pigs in both the animal-baited tent trap study and the feeding index calculation. However, any fitness advantage for *An. farauti* associated with feeding on humans in Haleta village is uncertain as the advantage of increased fecundity after feeding on human blood was offset by a longer time to oviposition. If larger blood meals are taken from human hosts, they may require a longer time to digest; as reported previously blood meal size is linked with increased fecundity [36–38].

The success of feeding on humans, the HBI, for *An. farauti* varies widely among the geographic areas where it has been measured. In Papua New Guinea, the HBI of females collected indoors was 0.65–1.00 [5, 39–41], when pooled for both indoors and outdoors was 0.68–0.88 [42, 43], and when collected outdoors was 0.07–0.85 [2, 5, 39–41, 44]. In the Solomon Islands, the HBI of females collected outdoors on Guadalcanal was 0.43 [45]. In this study in Central Province, the HBI (0.93) for *An. farauti* was higher than previous reports for outdoor resting collections. In villages where the outdoor HBI was low, the density of domestic animals was high and conversely in villages with a high outdoor HBI the density of alternative hosts was low [5, 39, 41, 44], as was the situation in Haleta where the numbers of domestic animals were scarce compared to humans. The influence of host availability in determining anthropophagy in *An. farauti* populations is particularly evident when the Haleta village population in the Solomon Islands is contrasted with the *An. farauti* population in Maraga village, Madang Province, Papua New Guinea, where pigs were abundant compared to humans and the HBI of *An. farauti* was 0.07. Consistent with the low HBI, the feeding index in Maraga Village, Papua New Guinea, indicated that *An. farauti* preferred pigs to humans (feeding index ranged from 3.37–6.80 across villages) [5]. Across the region where it is found, populations of *An. farauti* exist in isolation with restricted gene flow [46] and thus it is plausible that these populations are under different selective pressures for host selection. The observed differential feeding indices for *An. farauti* in the Solomon Islands and Papua New Guinea are consistent with the existence of isolated populations.

Fidelity of host selection for humans can have significant repercussions on the effectiveness of interventions that target human host feeding. If populations of mosquitoes are composed of subpopulations associated with different behaviours (such as host species preference, time or location of blood feeding [indoor/outdoor]), the impact of interventions to control malaria will differ than if the vector population is a single population. Host preferences of mosquitoes have a genetic basis [22, 25–27], and heterogeneous subpopulations were identified from mark-release-recapture experiments in some species (i.e. *An. balabacensis* in Malaysia [17–19], *An. minimus* in Thailand [20], *An. vestitipennis* in Mexico [21] and *An. gambiae* in Tanzania [22]) but not in others (e.g., anthropophagic and zoophagic subpopulations were not found for *An. maculatus* in Malaysia [47] and *An. culicifacies* in Sri Lanka [48]). If subpopulations of anthropophagic and zoophagic feeding mosquitoes do not exist, then even where the HBI is low (because of the presence of a large number of domestic animals, for example), vector control strategies targeting human blood feeding (such as insecticide treated nets or indoor residual spraying) can still be effective, as a significant proportion of the population will eventually seek a blood meal on a human during at least one feeding cycle during the period of the extrinsic incubation period.

## Conclusion

Successful blood feeding by mosquitoes is a function of a myriad of intrinsic and extrinsic factors that include the relative attraction to available host species with blood feeding on a host species having potential implications for reproductive fitness. *An. farauti* in the Solomon Islands was significantly (but slightly) more attracted to humans compared to pigs but human feeding was not unequivocally associated with a fitness advantage as the increased fecundity from human feeding was offset by a longer gonotropic cycle. *Anopheles farauti* fed predominantly on humans in the Solomon Islands and a strong determinant for the 0.93 HBI was the high relative abundance of humans compared to alternative hosts. Evidence was found for a single population of *An. farauti* with regards to host fidelity. Because *An. farauti* is a single population, interventions such as long-lasting insecticidal nets and indoor residual spraying that protect the human host will target the entire *An. farauti* population.

## Availability of data and materials

The datasets supporting the conclusions of this article are available in the James Cook University Tropical Data Hub repository: http://dx.doi.org/10.4225/28/56BD6DF7C9CB8.

## Additional file

10.1186/s12936-016-1168-y Graphical comparison of *Anopheles farauti* densities caught with human landing catch (HLC) and on barrier screens in Haleta village, Central Province, Solomon Islands.

## Abbreviations

FI: feeding index
GLM: generalized linear model
HBI: human blood index
HLC: human landing catch
