# Supplementary material for: Determinants of host feeding success by Anopheles farauti
Source: Malar J. 2016 Mar 10;15:152. doi: 10.1186/s12936-016-1168-y (PMC4785651; doi:10.1186/s12936-016-1168-y)
Supplement: Supplementary file 1 — 10.1186/s12936-016-1168-y Graphical comparison of Anopheles farauti densities caught with human landing catch (HLC) and on barrier screens in Haleta village, Central Province, Solomon Islands. [file 12936_2016_1168_MOESM1_ESM.pdf]

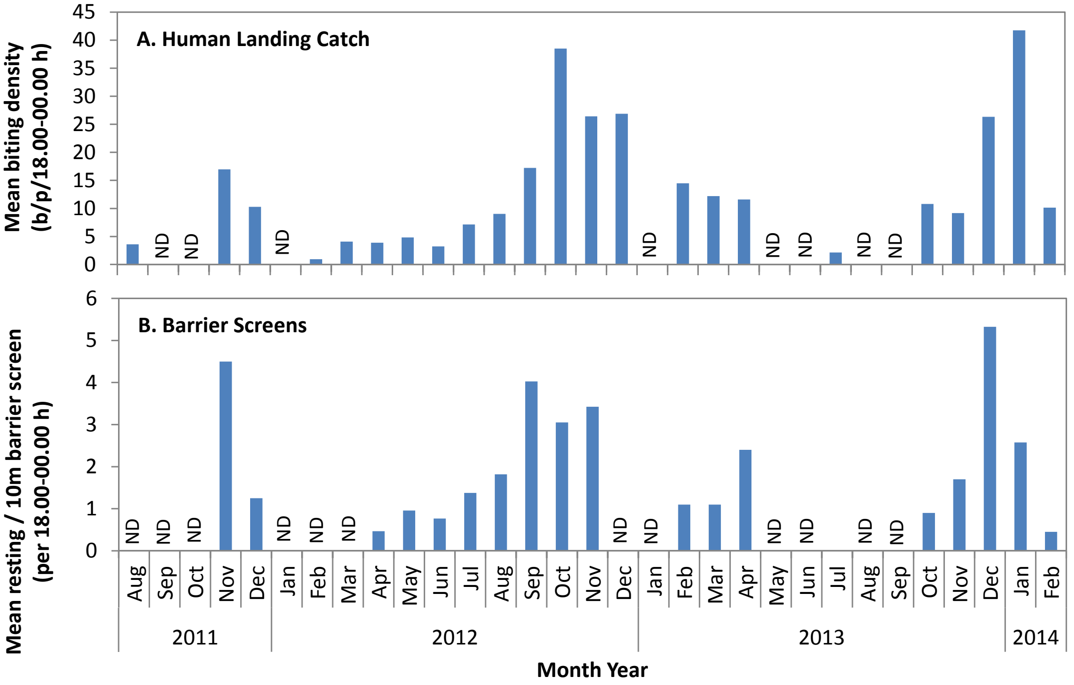

Additional file 1 Graphical comparison of *An. farauti* densities caught with human landing catch (HLC) and on barrier screens in Haleta village, Central Province, Solomon Islands.

Note: b/p/18.00-00.00 h = bites/person/18.00-00.00 h.
